# The Impact of Some Modulators of the Renin–Angiotensin System on the Scopolamine-Induced Memory Loss Mice Model

**DOI:** 10.3390/brainsci13081211

**Published:** 2023-08-16

**Authors:** Daniela-Carmen Ababei, Ioana-Miruna Balmus, Walther Bild, Alin Stelian Ciobica, Radu Marian Lefter, Răzvan-Nicolae Rusu, Gabriela Dumitrita Stanciu, Sabina Cojocaru, Monica Hancianu, Veronica Bild

**Affiliations:** 1 Pharmacodynamics and Clinical Pharmacy Department, Grigore T. Popa University of Medicine and Pharmacy, 16 Universitatii Street, 700115 Iasi, Romania; dana.ababei@gmail.com (D.-C.A.); razvan.nicolae.rusu@gmail.com (R.-N.R.); veronica.bild@gmail.com (V.B.); 2 Advanced Research and Development Center for Experimental Medicine (CEMEX), Grigore T. Popa University of Medicine and Pharmacy, 16 Universitatii Street, 700115 Iasi, Romania; gabriela-dumitrita.s@umfiasi.ro; 3 Department of Exact Sciences and Natural Sciences, Institute of Interdisciplinary Research, Alexandru Ioan Cuza University, 700506 Iasi, Romania; 4 Center of Biomedical Research, Romanian Academy, B dul Carol I, no 8, 700505 Iasi, Romania; alin.ciobica@uaic.ro (A.S.C.); radu_lefter@yahoo.com (R.M.L.); 5 Department of Physiology, Grigore T. Popa University of Medicine and Pharmacy, 16 Universitatii Street, 700115 Iasi, Romania; 6 Department of Biology, Faculty of Biology, Alexandru Ioan Cuza University, 700506 Iasi, Romania; sabina_18ro@yahoo.com; 7 Academy of Romanian Scientists, Splaiul Independentei nr. 54, Sector 5, 050094 Bucuresti, Romania; 8 Department of Pharmacognosy, Faculty of Pharmacy, Grigore T. Popa University of Medicine and Pharmacy, 16 Universitatii Street, 700115 Iasi, Romania; monica.hancianu@umfiasi.ro

**Keywords:** RAAS, cognitive functions, oxidative stress, Ramipril, Losartan, scopolamine, blood serum, brain, mice model, antioxidant effect

## Abstract

As some of the renin–angiotensin–aldosterone system (RAAS)-dependent mechanisms underlying the cognitive performance modulation could include oxidative balance alterations, in this study we aimed to describe some of the potential interactions between RAAS modulators (Losartan and Ramipril) and oxidative stress in a typical model of memory impairment. In this study, 48 white male Swiss mice were divided into six groups and received RAAS modulators (oral administration Ramipril 4 mg/kg, Losartan 20 mg/kg) and a muscarinic receptors inhibitor (intraperitoneal injection scopolamine, 0.5 mg/kg) for 8 consecutive days. Then, 24 h after the last administration, the animals were euthanized and whole blood and brain tissues were collected. Biological samples were then processed, and biochemical analysis was carried out to assess superoxide dismutase and glutathione activities and malondialdehyde concentrations. In the present experimental conditions, we showed that RAAS modulation via the angiotensin-converting enzyme inhibition (Ramipril) and via the angiotensin II receptor blockage (Losartan) chronic treatments could lead to oxidative stress modulation in a non-selective muscarinic receptors blocker (scopolamine) animal model. Our results showed that Losartan could exhibit a significant systemic antioxidant potential partly preventing the negative oxidative effects of scopolamine and a brain antioxidant potential, mainly by inhibiting the oxidative-stress-mediated cellular damage and apoptosis. Ramipril could also minimize the oxidative-mediated damage to the lipid components of brain tissue resulting from scopolamine administration. Both blood serum and brain changes in oxidative stress status were observed following 8-day treatments with Ramipril, Losartan, scopolamine, and combinations. While the serum oxidative stress modulation observed in this study could suggest the potential effect of RAAS modulation and scopolamine administration on the circulatory system, blood vessels endothelia, and arterial tension modulation, the observed brain tissues oxidative stress modulation could lead to important information on the complex interaction between renin–angiotensin and cholinergic systems.

## 1. Introduction

Despite that it is currently accepted that the essential role played by the renin–angiotensin–aldosterone system (RAAS) is in modulating blood pressure [1,2,3], it was showed that, in the brain, RAAS exerts a variety of effects associated with the cognitive functions with respect to memory, anxiety, and pain perception [4,5,6]. However, the functions of angiotensin II in memory processes remain highly controversial since Braszko et al. [7] demonstrated angiotensin II treatment has significant facilitatory effects in cognitive performance, while others have shown its negative outcomes or lack of effects on cognitive processes of both for angiotensin II and for some of its blockers [8,9,10], differences often attributed to behavioral tests variety, doses diversity, experimental designs, or animal models differences.

The correlation between RAAS modulation and other neuropsychiatric disorders was also proposed for mild cognitive impairment [11], Parkinson’s disease [12], anxiety and depression [3,6], autism [13], or schizophrenia [14,15]. In addition, recent evidence suggested that RAAS modulators interaction with β-amyloid metabolism and subsequent inflammatory processes could be a major component of Alzheimer’s disease [16]. 

**Figure 1:**
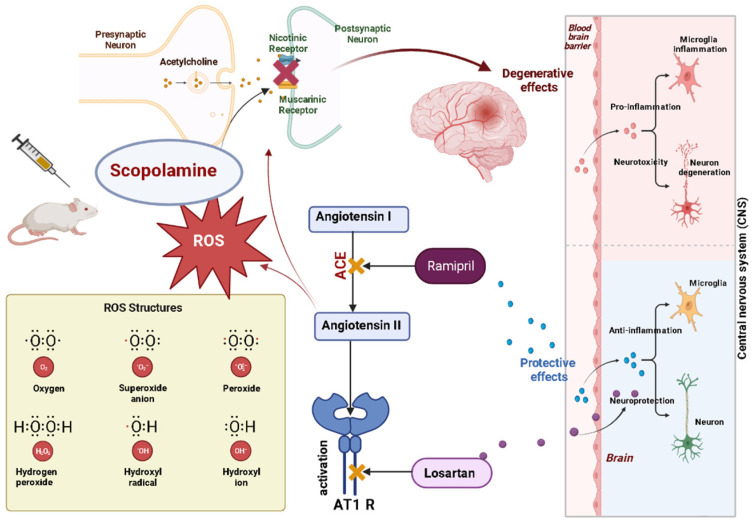
Renin–angiotensin–aldosterone system modulators and the implication of oxidative stress in cognitive processes.

Furthermore, the implication of oxidative stress in cognitive processes, brain molecular mechanisms, and RAAS was previously described, and we summarize it in Figure 1. It was shown that RAAS modulators could attenuate memory deficits by interacting with inflammatory and apoptotic markers [17]. Losartan (LOS) is a first-generation angiotensin I receptors antagonist activated by the liver which produces two of its metabolites, a competitive angiotensin I receptor blocker that prevents angiotensin II coupling to the mentioned receptor and an antioxidant factor that modulates mitochondrial oxidative functions [18]. Ramipril (RAM) is a potent angiotensin-converting enzyme inhibitor that significantly decreases the hepatic production of angiotensin II. RAM effects are further coupled with the decrease in angiotensin I and II receptors stimulation [19]. 

As some of the RAAS-dependent mechanisms underlying the cognitive performance modulation could include oxidative balance alterations, as previously showed in the hippocampus of angiotensin-II-intracerebroventricular-treated mice receiving an angiotensin-converting enzyme inhibitor [4], in this study we aimed to describe some of the potential interactions between RAAS modulators (RAM, LOS) and oxidative stress in a typical model of memory impairment (scopolamine—non-selective muscarinic receptors blocker). 

Scopolamine, a cholinergic neurotransmission blocker, causes both a decrease in neurotransmitter levels and the generation of ROS. All these effects can contribute to poor synaptic transmission, the initiation of inflammatory processes, impaired memory, cognitive decline, and finally, neuronal cell death. Activation of AT1 receptors of angiotensin II can generate a series of negative effects such as oxidative stress, with repercussions on cognitive status. The administration of Ramipril (ACEI) and Losartan (ABR) for various cardiovascular conditions can indirectly improve cognitive status due to the reduction in oxidative stress and anti-inflammatory effect.

## 2. Materials and Methods

### 2.1. Animals and Housing

The design of this study was previously described in [20] as a part of more complex experimental premises describing the neurobehavioral and molecular dynamics in the context of RAS modulation using enzymatic inhibitors and receptor blockers in a typical memory-loss mice model. 

Thus, for the evaluation of molecular changes in the context of RAS modulation, 48 white male Swiss mice with an average weight of 30 g were selected from the total experimental group and assigned for biochemical evaluation. Six homogenous groups of mice corresponding to the six experimental conditions, as described in our previous work on the neurobehavioral evaluation [20], were assigned for blood and tissues collection. Following a 15-day habituation period and behavioral analysis (radial maze test), the animals were euthanized according to the standard European and national regulations (using ketamine 100 mg/kg, xylazine 10 mg/kg).

During the full habituation period, experimental procedures, and tissue sampling, the animals were constantly supervised and evaluated by specialized personnel. Efforts were made to limit the number of animals used and their suffering. Romanian Laws on the animal experimentation in biomedical research and on animal health and welfare and the European Community Guidelines (Directive 2010/63/EU) were considered as gold standard in procedural guidelines of all the activities described in this study. Internal regulations of the study host institution (Grigore T. Popa University of Medicine and Pharmacy, Iasi, Romania) were also used as procedural guidelines in research, in accordance with the local ethics decision (from 6 October 2016).

### 2.2. Substances and Treatments 

**Figure 2:**
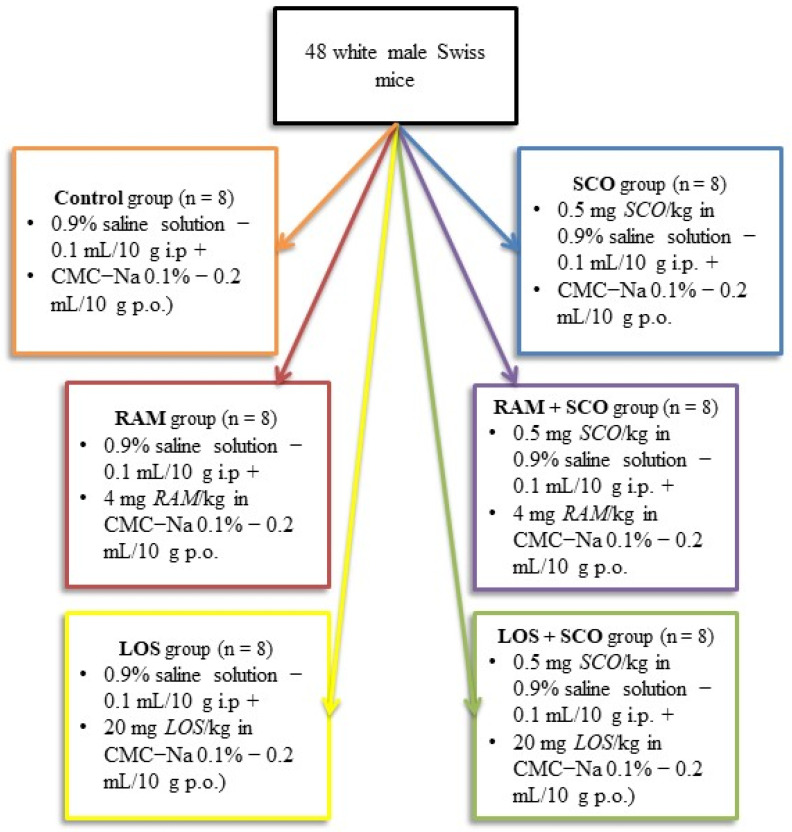
Schematic representation of the experimental design.

All the treatments and doses were carefully selected using the minimal effect rule while consulting the pharmaceutic recommendations and scientific literature previous reports on the neurobehavioral and molecular response of RAS modulation and scopolamine administration, as described in [20,21]. Detailed study groups and treatments description can be found in Figure 2. All the treatments were performed 30 min (SCO) and 1 h (RAM, LOS) prior behavioral assessment for 8 days.

### 2.3. Samples Collection and Preparation 

Biological samples—whole blood and brain tissues—were collected from the 48 animals at 24 h following the last chronic administration. Blood samples were allowed to form clots (by air exposure for 2½ h). Then, the clotted-blood-containing tubes were centrifuged at 3500 rpm for 15 min. Blood serum was separated into new tubes, aliquoted, and stored at −20 °C until further biochemical analysis. Brain tissues were extracted from the mice skulls, washed with saline, and used to obtain enzymatic extracts, as described by [22]. Then, 0.2 g brain tissues were thoroughly grounded and tissue extraction buffer was added in a 1:10 mass to volume ratio. The homogenate was centrifuged at 3500 rpm for 15 min and the supernatant was separated, aliquoted, and used for biochemical analysis. 

### 2.4. Biochemical Analysis

Total superoxide dismutase specific activity was determined using a Sigma Aldrich commercial 19160 SOD Determination Kit (19160-1KT-F Sigma Aldrich, Hamburg, Germany) according to the manufacturer’s instructions. SOD activity was indirectly measured using spectrophotometric based on the water-soluble formazan dye resulting from the water-soluble tetrazolium salt reaction with superoxide anion. Cellular glutathione peroxidase activity was determined using a Sigma Aldrich commercial kit (GPx Cellular Activity Assay Kit CGP-1, Sigma Aldrich, Hamburg, Germany) also by indirect determination based on the measurable nicotinamide adenine dinucleotide phosphate reaction media concentration decrease (consistent to the oxidation of NADPH to NADP+ during the GPx-mediated reduction of peroxides to water/alcohols). The specific enzymatic activity of SOD and GPx was calculated by reference to the total soluble protein content (determined using the total soluble protein Bradford assay, as previously described in [22]). 

Malondialdehyde concentrations were assessed using the thiobarbituric acid-reactive substances (TBARs) determination method, as described before [22,23]. Optimized volumes of blood serum/tissue extracts, trichloroacetic acid (50%), and thiobarbituric acid (0.73%) were mixed, vortexed, and incubated at 100 °C (water bath) for 20 min. Following cooling, 10 min centrifugation at 3000 rpm was performed. Similar to the biological samples’ reactions, an MDA standard curve was constructed using malondialdehyde bis (dimethyl acetal) (Sigma Aldrich, Hamburg, Germany) serial dilutions and was used for reference. The absorbances of the standards and samples were read at 532 nm against blank reaction. The results were expressed as mmol MDA/mL blood serum or mmol MDA/g tissue.

### 2.5. Statistical Analysis

All numerical data obtained following biochemical evaluation calculus were statistically analyzed using a Minitab 19 statistical tool (Pennsylvania University, Philadelphia, PA, USA, 2017). One-way analysis of variance (ANOVA) and one/two tailed *t*-test were performed for intergroup comparison. Intragroup comparison was verified for homogeneity using the Shapiro–Wilk normality test. Post-hoc analysis was performed using Tukey’s HSD test and Bonferroni corrected *t*-test. Statistical correlations were assessed using Pearson’s correlation and Spearman correlation. The results were expressed as means ± SEM and the intergroup differences and correlations were regarded as statistically significant when *p* < 0.05.

## 3. Results

### 3.1. The Effect of RAS Modulation in Scopolamine Induced Memory Loss on the Systemic and Central Nervous System Total Superoxide Dismutase Specific Activity 

**Figure 3:**
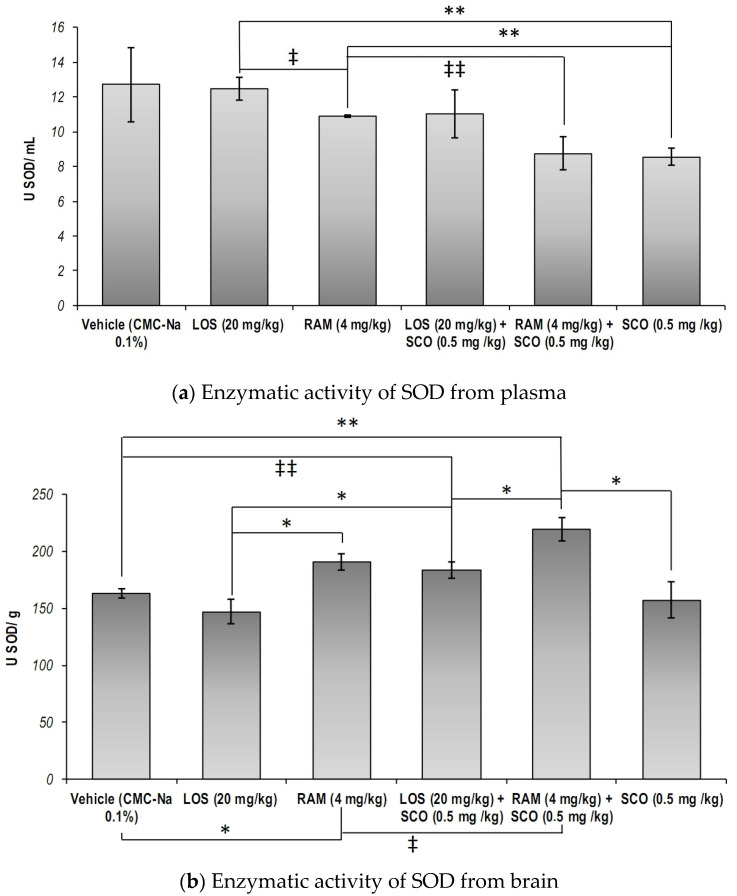
Total superoxide dismutase specific activity in blood serum and brain extracts in scopolamine-induced memory loss mice models treated with RAS modulators (CMC-Na = sodium carboxymethyl cellulose; LOS = Losartan; RAM = Ramipril; SCO = scopolamine). Results were expressed as means ± SEM (*n* = 8, for each group, (**a**)—** *p* < 0.01, ^‡^ *p* = 0.056, ^‡‡^ *p* = 0.06; (**b**)—* *p* < 0.05, ** *p* < 0.01, ^‡^ *p* = 0.06, ^‡‡^ *p* = 0.056).

The total SOD specific activity in the blood serum of the animals was decreased when SCO was administered, as compared to the control group (SCO versus Vehicle, *p* = 0.05, one tailed *t*-test). Despite that, our results showed no significant differences as a result of LOS and RAM treatments, as compared to the control group (Figure 3a, overall one-way ANOVA, *p* = 0.081). Furthermore, it was observed that both RAM and LOS treatments were not able to prevent SCO effects on the total SOD specific activity (RAM versus RAM + SCO, LOS versus LOS + SCO, SCO versus RAM + SCO, SCO versus LOS + SCO, *p* > 0.05). 

On the other hand, the analysis of the data obtained from brain tissues revealed that the total SOD specific activity revealed that RAM treatment could significantly increase total SOD specific activity, as compared to the control group (*p* = 0.014) and LOS treatment (*p* < 0.013) (Figure 3b, overall one-way ANOVA, *p* = 0.00077). A similar trend was observed following SCO administration in LOS and RAM pre-treated mice, as compared to the control group and respective positive controls, but with a lesser extent in RAM’s case (Control versus RAM + SCO, *p* = 0.002; RAM versus RAM + SCO, *p* = 0.06; Control versus LOS + SCO, *p* = 0.056; LOS versus LOS + SCO, *p* = 0.03). However, our data showed that RAM pre-treatment was better in modulating brain SOD specific activity in SCO-treated mice, as compared to the injury positive control (SCO), and LOS (RAM + SCO versus SCO, *p* = 0.016; RAM + SCO versus LOS + SCO, *p* = 0.032). 

### 3.2. The Effect of RAS Modulation in Scopolamine Induced Memory Loss on the Systemic and Central Nervous System Cellular Glutathione Peroxidase Specific Activity

**Figure 4:**
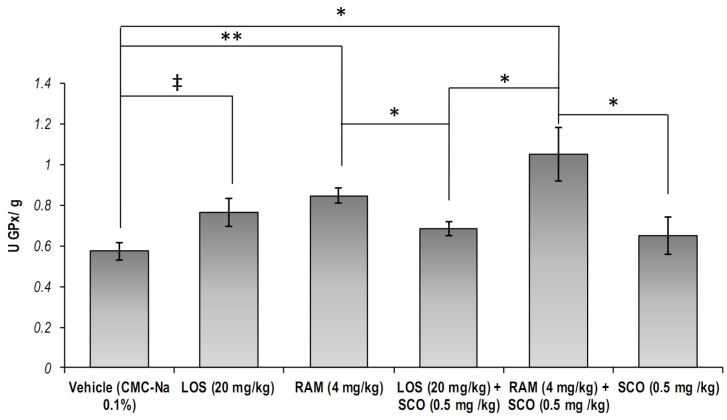
Cellular glutathione peroxidase specific activity in brain extracts in scopolamine-induced memory loss mice models treated with RAS modulators (CMC-Na = sodium carboxymethyl cellulose; LOS = Losartan; RAM = Ramipril; SCO = scopolamine). Results were expressed as means ± SEM (*n* = 8, for each group, * *p* < 0.05, ** *p* < 0.01, ^‡^ *p* = 0.051).

Significant differences of the GPx specific activity in the brain tissues were obtained (Figure 4, overall one-way ANOVA, *p* = 0.0047). We found that both RAM and LOS treatments could lead to increased cellular GPx specific activity, as compared to the control group (*p*_RAM_ = 0.002, *p*_LOS_ = 0.051). The pre-treatment with RAM in SCO-injured mice significantly prevented the impairment of GPx specific activity (RAM + SCO versus control, *p* = 0.014; RAM + SCO versus SCO, *p* = 0.047). Similarly, our results showed that RAM pre-treatment could better modulate GPx activity in SCO-treated mice, as compared to LOS pre-treatment (RAM + SCO versus LOS + SCO, *p* = 0.037).

### 3.3. The Effect of RAS Modulation in Scopolamine-Induced Memory Loss on the Systemic and Central Nervous System Malondialdehyde Concentration 

**Figure 5:**
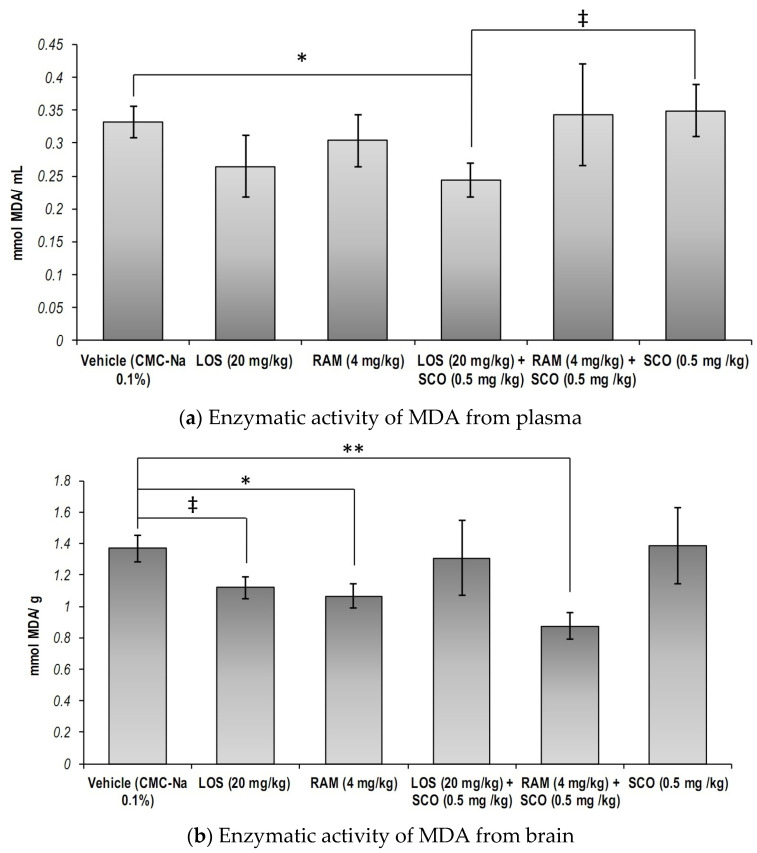
Malondialdehyde concentration in blood serum and brain extracts of the RAS-modulated scopolamine-induced memory loss mice models (CMC-Na = sodium carboxymethyl cellulose; LOS = Losartan; RAM = Ramipril; SCO = scopolamine). Results were expressed as means ± SEM (*n* = 8, for each group, (**a**)—* *p* < 0.05, ^‡^ *p* = 0.06; (**b**)—* *p* < 0.05, ** *p* < 0.01, ^‡^ *p* = 0.06).

Malondialdehyde concentrations within the biological samples were evaluated as a biomarker of lipid peroxidation [3]. We observed that neither in blood serum nor in the brain tissues did RAS modulation and SCO exposure lead to significant changes (Figure 5, overall one-way ANOVA, *p*_serum_ = 0.49, *p*_brain_ = 0.17). 

However, we noticed that systemic MDA concentrations were significantly decreased following SCO administration in LOS pre-treated mice, as compared to the control group (Figure 5a, LOS + SCO versus Control, *p* = 0.043), and had a suggestive trend as compared to the injury-positive control (LOS + SCO versus SCO, *p* = 0.06), leading to the assumption that the angiotensin II receptors blockage could prevent SCO-induced lipid peroxidation traceable from the blood sera. 

Regarding the brain lipid peroxidation, we observed that both RAM and LOS chronic treatments improved oxidative status, as suggested by the decreased MDA concentrations, as compared to the control group (Figure 5b, Control versus RAM, *p* = 0.036; Control versus LOS, *p* = 0.06), while RAM was better at preventing SCO-induced lipid peroxidation (RAM + SCO versus SCO, *p* < 0.05, one tailed *t*-test). Similarly, we observed that concomitant RAM and SCO exposure lead to significantly decreased MDA levels, as compared to the control group (Control versus RAM + SCO, *p* = 0.006).

### 3.4. Correlative Analysis of Molecular Biomarkers and Discussion on the Relevance of Systemic and Central Nervous System Oxidative Stress in RAS Modulation 

**Figure 6:**
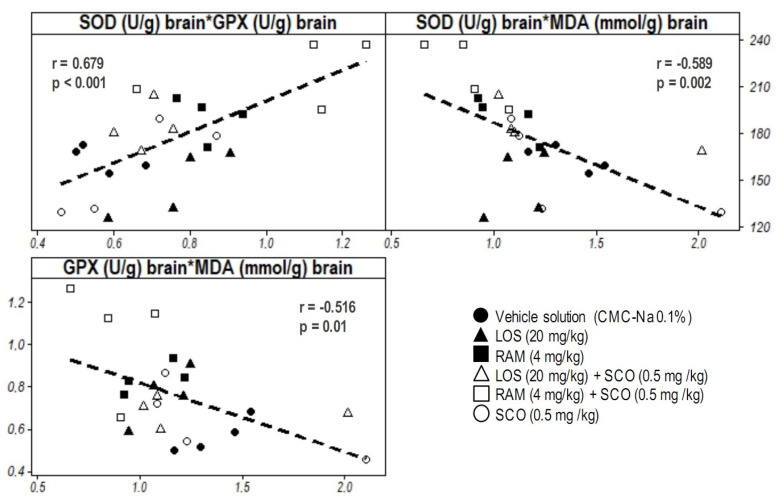
Correlative analysis of biochemical markers determined from brain extracts (CMC-Na = sodium carboxymethyl cellulose; LOS = Losartan; RAM = Ramipril; SCO = scopolamine). Results were expressed as means ± SEM (*n* = 8, for each group, Pearson’s correlation, r = Pearson coefficient, *p* = *p*-value).

The data analysis also revealed some possible correlations between the biochemical parameters evaluated from the brain tissues: moderately positive (SOD versus GPx, r = 0.679, *p* < 0.001) and moderately negative (MDA versus SOD, r = −0.516, *p* = 0.01; MDA versus GPx, −0.589, *p* = 0.002) (Figure 6). 

## 4. Discussion

In the present experimental conditions, we showed that RAS modulation via the angiotensin-converting enzyme inhibition (RAM) and via the angiotensin II receptor blockage (LOS) chronic treatments could lead to oxidative stress modulation in a non-selective muscarinic receptors blocker (SCO) animal model. In this context, our previous studies showed that both RAM and LOS could lead to significant changes in neurobehavioral landscape of animal models with or without muscarinic receptors modulation. In this way, we showed that RAM and LOS significantly enhanced short- and long-term memory [20], as well as promoted several affective behaviors improvement [2]. As our group previously described some memory impairments in RAS modulation and/or non-selective muscarinic receptors modulation [2], we are currently focusing on the oxidative changes occurring in blood serum and brain tissues to determine the possible response of the circulatory system and central nervous system.

Our previous studies showed that chronic SCO administration leads to systemic and brain oxidative stress promotion in a typical rodent model [2,24]. However, in this study, we showed that 8-day chronic intraperitoneal SCO treatment could not lead to significant oxidative stress in blood and brains of the tested mice. The current results suggested that chronic exposure to moderate intraperitoneal SCO doses could trigger an adaptative mechanism in which the metabolism slowly and gradually counteracts SCO effects. While this mechanism was initially described by Marks et al. [25] and Kohl et al. [26] in behavioral contexts by altering cholinergic metabolism, we are currently observing it in a molecular context.

This study also brings reasonable evidence that both RAS and non-selective muscarinic receptors modulation could lead to oxidative balance modulation. Thus, our results suggested that systemic and brain oxidative status modulation could be obtained through angiotensin-converting enzyme inhibition (RAM administration), angiotensin II receptors blockage (LOS administration), non-selective muscarinic receptors blockage (SCO administration), and their combination. In this experimental context, we observed that the different variants of RAS modulation could boost or reduce the cholinergic receptors blockage effects. The interaction between RAS and the cholinergic system was previously described by [27] in ovine fetuses in relation to cardiovascular signaling in heart activity modulation and by [28] who recently described this interaction together with oxidative stress implication in the context of Alzheimer’s disease and other neurodegenerative diseases. 

Several recent studies showed that RAM and LOS could exhibit antioxidant properties [29,30]. Our results also confirmed their findings regarding SOD activity modulation (increased in blood serum), and we observed that LOS could prevent SCO-induced systemic lipid peroxidation. This antioxidant, anti-ischemic, and anti-apoptotic potential of LOS was previously showed by [31] in a study on the blood vessels endothelial oxidative damage effects. However, in some cases, SCO was shown to potentiate the first line antioxidant enzymatic defense; thus, in the context of the modulatory interaction between choline’s and angiotensin receptors [32], the RAS modulation studies showed that salinity plays an important role in the angiotensin II receptors blockage by muscarinic mechanisms [1,2]. In this way, we observed that the extent of the RAS modulation on the total SOD specific activity in brain tissues mainly depends on the modulation mechanism (angiotensin-converting enzyme inhibition versus angiotensin receptor blocking). However, the non-selective muscarinic receptors blockage (through SCO administration) could lead to significant modulation of oxidative stress even following 8-day treatments with RAS modulators. 

Regarding the LOS facilitatory effect against oxidative stress, we observed that oxidative modulation was significant in brain tissues, as compared to blood serum, in our animal model. In the cardiovascular system, LOS could prevent angiotensin II binding to the subsequent receptor resulting in arterial pressure decrease and blood vessels constriction [33]. Due to LOS effects on the blood–brain barrier permeability modulation, as [34] showed in a rat model of angiotensin-II-induced arterial hypertension, LOS could also exhibit modulatory effects on the brain tissues. Thus, we previously showed that RAS modulation using LOS and RAM could improve cognitive performances and socio-affective behavior, while Abiodun and Ola [35] and Campos and Pacheco [36] provided an updated perspective of the RAS system in neurodegeneration and inflammation. Moreover, Ahmed et al. [37] and Mirzahosseini et al. [38] even described promising mechanisms in which RAS modulation could prevent progressive cognitive impairments and inflammation following brain-injury-derived neurodegeneration. An intimate implication of RAS systems with oxidative stress was also suggested by [34], describing the pathway through angiotensin II that could modulate nitric oxide metabolism. 

Our results also suggested that RAS system modulation via angiotensin II receptors inhibition could prevent oxidative stress damages occurring in scopolamine-induced neurodegeneration. De-Hua He et al. [39] extensively explained the correlation between LOS, angiotensin II, and oxidative stress in a complex study on the cerebral lesions occurring in a rat model of hypertension-induced brain ischemia. Since one of the most important processes that modulates apoptosis is oxidative stress, it could be suggested that LOS could potentially exhibit protective effects against cerebral tissues oxidative stress. Thus, we observed the significant antioxidant potential of LOS as suggested by the important increase in cellular GPx activity, significantly correlated with lipid peroxidation intensity decrease through its potential to catalyze the reduction of hydrogen peroxide (most abundant ROS) and organic peroxides residing in the lipid-rich cellular membranes. Similar results were reported by Saavedra et al. [40,41] that are also discussing the antioxidant potential of LOS alongside its neuroprotective effect in the context of stress coping mechanisms, anxiety, inflammation, and ischemia. Despite these, the most promising antioxidant potential of LOS was demonstrated in its effects on preventing heart muscles atrophy [42], fibrosis [43], and hypertrophy [44]. Nevertheless, these studies discuss the LOS potential to prevent brain lesions caused by oxidative stress, as also suggested by our results in the condition of concomitant blockage of angiotensin II receptors and muscarinic receptors. 

Similarly, a mild antioxidant effect of RAM was suggested by our results. RAM physiologic effects include the inhibition of angiotensin II converting enzyme and the inhibition of lipid peroxidation via antioxidant enzymatic defense potentiation (SOD enzyme activity modulation) [45]. Our results are confirming that SOD activity in brain tissues could be independent on the non-selective muscarinic receptors blockage. Moreover, while RAM could modulate cellular GPx specific activity, future studies could evaluate the cause–effect interplay within the interaction between RAS modulation and the non-selective muscarinic receptors blockage by relation to oxidative stress. For instance, the potential of RAM to modulate oxidative stress was argued by [46] in a diabetes animal model while studying the effect of oxidative status on the vascular endothelium. Moreover, the antioxidant effect of this angiotensin-converting enzyme inhibitor was widely discussed by [47] in a comparative study of angiotensin-converting enzyme inhibitors effects on stroke frequency suggesting that angiotensin-converting enzyme inhibitors could be implicated in apoptosis via nitric oxide synthase modulation. Likewise, the positive effect of RAM has been demonstrated in other areas of research, such as the study of its effects on cognitive performance [48,49]. In these studies, some correlations between cognitive performance and oxidative status in the context of RAM treatment were showed in both physiological conditions and in some neuropsychiatric diseases [50]. Therefore, our data could further suggest that RAM could not only modulate the systemic oxidative status and the physiological processes related to angiotensin II activity, but also the oxidative status in the brain tissues, as its potential to modulate cellular apoptotic processes while decreasing the accumulation and effects of reactive oxygen species was previously showed. Moreover, while exploring the effects of RAM on the brain oxidative status in the context of non-selective muscarinic receptors blockage we found that RAM could exhibit protective potential against the SCO pro-oxidant effect. However, the interaction between RAM and SCO resulted in greater potentiation of antioxidant processes. These results can be argued by the activity and chemical properties of RAM, as the time between RAM pre-treatment and SCO and the relatively short half-life of the muscarinic receptor blocker allowed the much more active RAM-derived compound activation. 

A potential limitation of this study should be addressed: the pro-oxidant effects of scopolamine were seemingly in an incipient stage and not very relevant, which may be the result of the relative short scopolamine exposure duration given the low dosage of 0.5 mg/kg. Additional assays of catalase or other antioxidant enzymes activity might bring clarity to the oxidative dynamics and should be considered in future studies. Immunohistochemical analysis of different neuronal markers, RT-PCR, and western blot of ACE and AT2 could be performed in similar treated groups to provide a clearer perspective on the oxidative status. However, memory impairment in the present animal models was behaviorally confirmed in vivo in our previous preliminary study [20], which suggests the effectiveness of scopolamine in blocking cholinergic muscarinic receptors. 

## 5. Conclusions

RAS modulation and non-selective cholinergic receptors blockage could lead to oxidative status modulation. Both blood serum and brain changes in oxidative stress status were observed following 8-day treatments with RAM, LOS, SCO, and combinations. While the serum oxidative stress modulation observed in this study could suggest the potential effect of RAS modulation and SCO administration on the circulatory system, blood vessels endothelia, and arterial tension modulation, the observed brain tissues oxidative stress modulation could lead to important information on the complex interaction between renin–angiotensin and cholinergic systems. Our results showed that LOS could exhibit a significant systemic antioxidant potential partly preventing the negative oxidative effects of SCO and a brain antioxidant potential, mainly by inhibiting the oxidative stress-mediated cellular damage and apoptosis. RAM could also minimize the oxidative-mediated damage to the lipid components of brain tissue resulting from SCO administration.

## Data Availability

Data is contained within the current article.
